# Partial Fat Substitution in Butter Cookies Using Thermochemically Modified Unripe Plantain (*Musa paradisiaca* AAB) Flour Treated with Fumaric Acid

**DOI:** 10.1007/s11130-026-01545-6

**Published:** 2026-08-13

**Authors:** Jhon Esteban Armenta-Roncancio, Natalia Jose Muentes-Arismendy, Eduardo Rodriguez Sandoval, Jesus Humberto Gil-Gonzalez, Leonardo Alexis Alonso-Gomez

**Affiliations:** 1 Present Address: Facultad de Ciencias Agrarias, Departamento de Ingeniería Agrícola y Alimentos, Universidad Nacional de Colombia - Medellín Campus, Medellín, Colombia; 2 Facultad de Minas, Departmento de Procesos y Energía. Semillero de Investigación en Ciencia y Tecnología de Alimentos (SICTAL), Universidad Nacional de Colombia – Medellín Campus, Av. 80 #65 - 223, Villa Flora, Medellín, Colombia; 3 Present Address: Universidad de los Llanos, Grupo de Investigación “Ciencia, Tecnología e Innovación Agroindustrial (CITIA)”, Km. 12 Vía Puerto López, Villavicencio, Colombia

**Keywords:** Low-fat foods, Food analysis, Bakery products, Food processing, Modified flour

## Abstract

This study evaluated thermochemically modified unripe plantain flour treated with fumaric acid as a fat substitute in butter cookies. Incorporating this modified flour with a 24.73% fat reduction increased sensory acceptability by 65% compared with native unripe plantain flour. Fat reduction caused changes in mass loss during baking and porous structure while maintaining stable ash and protein contents and a higher proportion of nondigestible carbohydrates. Enzymatic hydrolysis analysis confirmed higher resistant starch content and lower proportions of rapidly and slowly digestible starch than in the control cookies. Thermochemically modified unripe plantain flour treated with fumaric acid proved to be a promising alternative for developing reduced-fat butter cookies by improving sensory acceptability, textural properties (increased crispness), and functional potential through reduced product digestibility. Reduced-fat cookies exhibited greater firmness and crispness but also greater browning intensity.

**Supplementary Information:**

The online version contains supplementary material available at https://doi.org/10.1007/s11130-026-01545-6.

## Highlights

The incorporation of thermochemically modified unripe plantain flour treated with fumaric acid enabled a 24.73% fat reduction in butter cookies while maintaining structural integrity.

Fumaric acid treatment increased sensory acceptability by 65% compared with cookies prepared with native plantain flour, effectively overcoming the typical sensory limitations associated with native plantain flour.

Formulated cookies exhibited a higher resistant starch content and lower rapidly and slowly digestible starch fractions, enhancing their potential as a functional food.

Despite fat reduction, the use of modified plantain flour improved the textural properties of cookies, resulting in greater firmness and crispness.

The use of thermochemically modified plantain flour reduced moisture loss during baking and produced a highly stable porous network, suggesting that fumaric acid stabilizes the starchy matrix during baking.

**Supplementary Information:**

The online version contains supplementary material available at https://doi.org/10.1007/s11130-026-01545-6.

## Introduction

Globally, there has been growing interest in reducing fat consumption in processed foods as a public health priority. Considering the high consumption of bakery and confectionery products, this sector of the food industry has become one of the main focuses of research [1].

For decades, epidemiological research has established a correlation between high fat consumption, especially saturated fat intake, and an increased risk of developing diabetes mellitus and cardiovascular disease [2]. Given this scientific evidence and consumers’ greater health awareness [3], the market has experienced two main trends: reducing fat in processed products, with a potential negative impact on sensory quality, and replacing fats with substitutes that have acceptable sensory characteristics [1, 4]. Mitigating the obesity epidemic requires reducing excessive sugar and fat intake [2].

Because of their technological characteristics, sensory properties, and prolonged shelf life, cookies are a common food in daily diets and are widely consumed by people of all ages. However, many commercial cookies contain low levels of dietary fiber and high proportions of saturated and *trans* fatty acids [5]. In cookie manufacturing, fats such as margarine, butter, and industrial fats obtained through the hydrogenation of oils are commonly used. This process generates *trans* fatty acids, which are associated with increased cholesterol levels and the development of coronary heart disease [6]. Nevertheless, fats improve product texture, flavor, and shelf life. Therefore, reducing or replacing fat in baked goods could benefit consumer well-being and health [7].

The technological challenges of fat reduction in cookies primarily involve textural properties, including fracturability, softness, and moisture retention capacity [8]. Additionally, fats contribute to the dispersion of dough ingredients, affect dough plasticity, and promote structural stabilization during baking [9]. From a sensory perspective, fat must be replaced with substitutes that do not adversely affect mouthfeel, a determining factor in consumer acceptability [1].

Various fat substitution strategies have been developed and are mainly divided into complex carbohydrates and gums/gels. Among complex carbohydrates, enzymatically modified cassava starch has been incorporated as a fat substitute in crackers [4]. Among gums and gels, Punia and Dhull [10] used chia mucilage as a fat substitute because of its high water-holding capacity and ability to increase viscosity. Cui et al [11] developed gels with chitosan in the aqueous phase and monolaurin, medium-chain triglycerides, and cinnamaldehyde in the oil phase, obtaining lower viscosity than commercial fats while maintaining similar texture. These studies emphasize the use of plant-derived functional ingredients as fat substitutes, including modified carbohydrates, gels, and gums. Within this framework, plantain (*Musa paradisiaca* AAB) is a promising candidate because of its high starch content. It is a widely cultivated tropical crop and a staple food in various regions of Africa [12], Asia [13], and the Americas [14]. Its unripe pulp is processed into a flour rich in resistant starch (RS) and micronutrients (potassium and phosphorus), with considerable potential for food applications [15]. However, these flours often do not meet the technological and functional requirements of various food formulations in their native state [16]. Their composition must therefore be modified through physical, chemical, or enzymatic processes, or combinations thereof (dual or multiple modifications), to confer new functional attributes and enhance their technological value [17], as demonstrated by Rahmadani et al [18], who described the development of RS properties in foods through treatment with organic acids.

However, native unripe plantain flour exhibits limited functional properties [19]. Physicochemical or enzymatic modifications are used to improve characteristics such as swelling, solubility, and retrogradation, with chemical treatment representing a common strategy [20]. The effectiveness of these modifications depends on parameters such as pH, time, temperature, and the modifying agent used [21, 22]. The interaction of the starch present in native unripe plantain flour with acids produces modifications through hydrolysis [23], esterification [17], or cross-linking [21, 24], thereby altering the physicochemical and functional properties of the starch in the flour. These reactions take advantage of the susceptibility of the amorphous structure, particularly the amylose segments [24], and the lamellae of semicrystalline starch. The introduction of acid into the starch structure affects both the particle surface and its internal regions [25].

Among the acids capable of producing such modifications, fumaric acid (E-297) stands out as a versatile dicarboxylic agent widely used in the food industry. It is employed as an acidulant, flavor enhancer [26], preservative, leavening improver in baking [27], and dough conditioner in breadmaking [28]. Its notable characteristics include low toxicity, low hygroscopicity [29], high acidifying power [30], and high acid dissociation constants, which confer high efficiency as a functional agent [31]. Additionally, it readily participates in esterification reactions [21, 24]. These properties improve key characteristics such as water- and fat-holding capacity, as well as flour solubility and digestibility, making fumaric acid a valuable tool for developing functional formulations.

Although studies have investigated the use of fruit flours as functional ingredients in breadmaking [32] and fat reduction in cookies through alternative ingredients [33], no studies have been identified that combine these approaches by using thermochemically modified unripe plantain flour treated with fumaric acid. The closest precedent is the study by Sánchez et al [17], who produced cookies using plantain flour esterified with citric acid, a tricarboxylic acid. However, that study differs from the present work in two fundamental ways: first, it used different heat treatments during modification; second, the modified flour was used to replace wheat flour (the carbohydrate fraction), whereas in the present study it was used as a fat substitute.

This study evaluated the effect of partially replacing butter with thermochemically modified unripe plantain flour treated with fumaric acid on the physicochemical and sensory characteristics of reduced-fat butter cookies.

## Materials and Methods

The Materials and Methods section is presented as supplementary material.

## Results and Discussion

### Physicochemical Properties of Cookies

The physicochemical characteristics of baked products prepared with three treatments were evaluated: Control (wheat flour with a conventional fat formulation), T1 (fat-reduced cookies formulated with native plantain flour), and T2 (fat-reduced cookies formulated with thermochemically modified plantain flour). A significant difference (*p* < 0.05) was observed in mass loss during thermal processing. The control product exhibited the greatest mass loss, whereas products containing plantain flour showed significantly lower losses, with no significant differences between T1 and T2 (*p* > 0.05). This lower mass loss in products containing plantain flour, especially T2, suggests greater water-holding capacity within the structural matrix of the dough, thereby reducing moisture loss during baking. This effect can be attributed to the incorporation of plantain flour, which contributes water-holding capacity, as reported by Jiménez and Navarro [34]. By contrast, the greater mass loss observed in the control product may be attributable to its higher fat content and greater loss of volatile compounds.

Product thickness was significantly lower (*p* < 0.05) in T2 than in the control and T1 products, with no significant difference between the latter two (*p* > 0.05). This finding indicates that treatment with modified plantain flour affected the final thickness of the product. Regarding the expansion factor, T2 exhibited a significantly higher value (*p* < 0.05) than the control product and T1, with no significant difference between the latter two (*p* > 0.05). This increase in the expansion factor of T2 could indicate greater release or migration of water vapor and gases during thermal processing, thereby contributing to a larger surface area [35].

Despite its higher expansion factor, T2 exhibited a significantly lower apparent volume (*p* < 0.05) than the control product and T1, with no significant difference between the latter two (*p* > 0.05). Although the surface expanded, the microstructure of T2 was more compact and less porous, resulting in a lower overall volume. This behavior differs from that reported by Milićević et al [36] in fat-substitution studies of bakery products and may indicate specific effects of thermochemically modified unripe plantain flour treated with fumaric acid. The lower porosity of T2, inferred from its lower apparent volume, could be due to structural modification of plantain starch that affected its gelatinization capacity and/or the formation of a protein-starch matrix during thermal processing [18, 27].

Additionally, moisture content and water activity ($\:{a}_{w}$) were compared among the different samples. Moisture content was significantly higher (*p* < 0.05) in T1 than in the control product and T2, with no significant difference between T2 and the control product (*p* > 0.05). This finding indicates that native plantain flour favored water retention in the final product. These results are similar to those reported by Sánchez-Rivera et al [17] for bakery products prepared with citric acid-modified starches, in which the addition of citric acid also influenced moisture content. Regarding water-holding capacity, no significant differences (*p* > 0.05) were observed among treatments, consistent with the findings of Jeong et al [37]. This suggests that the lower mass loss observed in T2 is not necessarily due to a higher intrinsic water-holding capacity of the flour but rather to a more stable structural matrix formed during baking that physically entraps moisture or more effectively limits its evaporation than in the other treatments [35].

Regarding water activity ($\:{a}_{w}$), significant differences (*p* < 0.05) were observed among all treatments. T1 showed the highest value, followed by the control product, whereas T2 exhibited the lowest value. These results, although slightly higher than those reported by Suo et al [38] for legume-based bakery products with reduced sugar and lipid contents, suggest that native plantain flour increases the availability of free water in the product, whereas thermochemically modified unripe plantain flour treated with fumaric acid decreases it. The reduction in $\:{a}_{w}$ observed in T2 could be due to greater interaction of water with modified RS structures, making it less available, which is beneficial for the microbiological stability of the product.

### Proximate Composition of Cookies

In the baked products prepared with the different treatments, several chemical parameters were evaluated, including dough pH and the contents of protein, fat, and ash, all of which are relevant indicators of the nutritional and functional quality of the products. Proximate composition analysis showed that protein and ash contents did not differ significantly (*p* > 0.05) among treatments. This indicates that partial fat substitution with plantain flour did not significantly affect the protein or mineral content of the final formulations.

The proximate composition was similar among the products analyzed in this study, which is consistent with the findings of García-Solís et al [5], who investigated the use of unripe plantain flour in gluten-free bakery products rich in dietary fiber by comparing it with standard RS. Products prepared with native plantain flour (T1) and modified plantain flour (T2) achieved an experimental reduction in fat content of approximately 22.67% compared with the control product (Table S1), decreasing from 30.26 g/100 g in the control to approximately 23.40 g/100 g in the substituted treatments. These experimental values differ slightly from the theoretical butter reduction of 24.73% (w/w) calculated from the initial formulation mass balance. This minor discrepancy is expected in baked matrices because theoretical mass balances often do not account for the formation of nonextractable carbohydrate-lipid and protein-lipid complexes during dough mixing and baking, which can lead to minor deviations in Soxhlet or acid-hydrolysis quantification [1].

Nevertheless, this reduction is substantially greater than that reported by Kwon and Chang [39] for fat substitution in bakery products using rice flour modified by citric acid esterification. The acidic modification of the flour used in T2 caused a significant decrease (*p* < 0.05) in dough pH. This result is expected because of the acidic nature of the fumaric acid used during thermochemical modification, which is retained in the dough matrix.

### Fracturability

In this context, a greater force (N) required to break the product indicates lower fracturability (*i.e*., greater firmness). The addition of plantain flour and fat reduction significantly increased product firmness (*p* < 0.05), requiring considerably greater force to induce fracture. The order of fracture resistance was T1 > T2 > Control. These results are consistent with those reported by Patiño-Rodríguez et al [40], who found that increasing the proportion of plantain solids increased the firmness of extruded spaghetti. Additionally, these findings suggest that thermochemical treatment with organic acids in T2 altered the structure and molecular interactions of the native flour. This observation is consistent with Armenta et al. [41], who reported that thermochemical modification with fumaric acid significantly influences the functional and structural characteristics of plantain flour components. Similarly, the esterification of corn starch with malic acid through heat treatment has been documented [24]. These observations reinforce the conclusion that thermochemical modification with fumaric acid is an effective means of producing functional plantain flour that can partially replace fat while maintaining the rheological, sensory, and textural attributes of reduced-fat shortbread cookies.

This textural behavior is associated with greater crispness, a generally desirable sensory characteristic that correlated positively with sensory acceptance [42]. By contrast, the control products, although requiring less force to fracture, exhibited a different texture. According to García-Solís et al [5], protein-starch interactions stabilized by hydrogen bonds play a key role in determining the textural properties of low-moisture products such as baked goods. In the reduced-fat products, in which plantain flour acted as a fat substitute, the limited development of the gluten network resulting from the absence of kneading, together with the lower butter content, produced a harder structural matrix [43].

### Color

The addition of plantain flour significantly altered product color (*p* < 0.05) compared with the control sample (without plantain flour). Specifically, products containing plantain flour exhibited significantly lower lightness (L*) (*p* < 0.05), indicating a darker color than the control. Additionally, they showed a slightly redder hue (a*) (*p* < 0.05) and a significantly more yellow hue (b*) (*p* < 0.05). The T1 and T2 samples exhibited the greatest color intensity. This decrease in lightness and increase in yellowness were reflected in lower whiteness index values for T1 and T2 than for the control product, which had the highest WI. These results agree with those of Sánchez-Rivera et al [17], who reported that bakery products made with citric acid-esterified plantain flour received lower aesthetic ratings from consumers than wheat flour products because of perceptible color differences. This finding underscores the importance of color in consumer choice and acceptance. Moreover, chromaticity (C*) was significantly higher (*p* < 0.05) in products containing plantain flour, indicating a more saturated color than in the control product. Similar results were reported by Pasqualone et al [44] when comparing corn flour-based bakery products with wheat flour products. Taken together, these findings indicate that the addition of plantain flour altered the chromatic profile of the products, a critical factor in consumer sensory perception.

### *In Vitro* Digestibility and Starch Digestion Kinetics of Cookies

Evaluation of in vitro starch digestibility has attracted growing interest in the exploration of new starchy sources because it reflects the resistance or delay of starch hydrolysis by α-amylase and amyloglucosidase [21, 24]. This approach makes it possible to evaluate the behavior of rapidly digestible starch (RDS), slowly digestible starch (SDS), and RS fractions relative to total starch and the sugar content inherent to the product matrix.

Total starch content and reducing sugar content did not differ significantly (*p* > 0.05) among the different product formulations (Control, T1, and T2). However, the incorporation of plantain flour had a notable effect on starch digestion kinetics.

Specifically, a reduction in the proportion of RDS (t < 20 min) was observed, following the order T1 < T2 < Control. This finding is consistent with the review by Suo et al [38], who also reported that starch modification tends to decrease both the RDS and SDS fractions. Similar results have been reported in studies of modified plantain flours by Koteswara Reddy et al [22] and in bakery products prepared with plantain flours by Sánchez-Rivera et al [17]. These findings suggest a consistent effect of plantain starch modification on its in vitro digestibility profile. The incorporation of modified plantain flour as a fat substitute significantly increased the proportion of RS. Beyond its technological role in fat reduction, this flour demonstrated functional value by increasing the proportion of solids resistant to enzymatic hydrolysis.

This increase suggests that modification of plantain flour reduces starch digestibility while enriching the RS fraction of the products. Consequently, the use of plantain flour offers a dual advantage: reduced fat content and an increased RS fraction. These results agree with previous studies of products containing lintnerized plantain starch [5, 45] and citric acid-esterified plantain flour [17].

### Starch Hydrolysis Kinetics and Modeling

The graphical representation of starch digestion (Supplementary material) illustrated the variation in the amount of digested starch over time. Analysis of the digestion kinetics revealed a consistent trend: the amount of digested starch increased progressively as digestion time increased, as reported by Olawoye et al [23]. However, the accumulation of digested starch varied among the product formulations evaluated. To model the starch digestion kinetics of the developed products, a first-order model was used to describe the progression of starch digestion. Specifically, the kinetic constant, which reflects the rate of the enzymatic reaction, showed a decreasing trend in the order Control > T1 > T2. This pattern suggests that the rate of starch digestion was influenced by the specific composition of each product formulation. Consistent with the findings of Luo and Kim [46], the increasing interaction between the substrate—the starch present in the product—and the digestive enzymes (α-amylase and amyloglucosidase) as digestion progressed was a key factor explaining the observed increase in the amount of hydrolyzed starch.

Likewise, the lower D(∞) value observed in products made with modified flours was consistent with that reported by Kembabazi et al [47] in their study of type V RS formation through treatment with organic acids, as well as with the findings of Wang et al [24] and Fernández-Almeida et al [48]. These authors reported that chemical and structural modifications of starch reduce its susceptibility to enzymatic hydrolysis. The T2 formulation consistently yielded the lowest values for the kinetic constant and the sum of squares, along with intermediate standard error values. These findings suggest that the formulation containing modified plantain flour delays starch digestion more effectively, a characteristic that may contribute to a lower glycemic index [2, 47].

### Sensory Acceptance

Frequency distribution analysis of sensory acceptance (Supplementary material) using Pearson’s chi-square test ($\:{\chi\:}^{2}$) revealed highly significant differences among treatments (*p* < 0.001; $\:{\chi\:}^{2}$ = 126.67; *df* = 4). This result confirms that the type of flour incorporated influences consumer preference, thereby rejecting the null hypothesis that sensory acceptance is independent of treatment.

The sample prepared with native plantain flour (T1) had the highest rejection rate, with 72.22% (65/90) of responses in the “I don’t like it” category. This behavior can be explained by the intrinsic properties of green plantain flour, whose dense structure and phenolic compounds contribute a characteristic astringency that limits its acceptability without prior treatment [49]. Meanwhile, the control sample showed a tendency toward neutrality, with 66.67% (60/90) of responses in the “Indifferent” category, suggesting an acceptable formulation but with limited sensory impact.

By contrast, thermochemical modification with fumaric acid significantly reversed the rejection trend observed for the native flour, with T2 achieving the highest acceptance rate at 61.11% (55/90) in the “I like it” category. This improvement was statistically greater than the expected frequency (E = 30), demonstrating that the modification not only enhances the functional properties of the starch but also effectively masks the undesirable sensory attributes of the fruit [5, 23, 42, 48].

Unlike the findings reported by Sánchez-Rivera et al [17], who reported low acceptance of bakery products made with citric acid-esterified flour, the results of the present study position T2 flour as a highly acceptable functional ingredient. Its ability to maintain an attractive sensory profile and favorable texture, even with reduced fat content, is demonstrated by the lowest rejection rate (16.67%) among all treatments.

## Conclusions

This study demonstrates that thermochemical modification with fumaric acid transforms unripe plantain flour into an effective functional ingredient for the reformulation of reduced-fat bakery products, overcoming the technological and sensory limitations historically associated with native plantain flour.

To our knowledge, this is the first study to systematically evaluate the combined effects of thermochemical modification with fumaric acid and fat reduction on the physicochemical, sensory, and nutritional properties of cookie-type bakery products. The findings demonstrate that this chemical modification strategy enables up to 24.73% fat substitution without compromising sensory acceptability, substantially outperforming native flour while highlighting specific interactions between fumaric acid and plantain starch that differ from those reported for other modified starch systems.

The principal functional contribution of this study lies in its dual benefit: the modified flour acts simultaneously as a technological fat substitute and as a source of low-digestibility starch, significantly increasing the RS fraction while reducing the rate of enzymatic hydrolysis. This improved nutritional profile makes the modified flour a promising ingredient for the development of foods with glycemic modulation potential.

Future research should focus on in vivo validation of the glycemic response, evaluation of oxidative stability and shelf life, and investigation of synergistic combinations with physical or enzymatic modification methods to further optimize the functional properties of the ingredient.

## Supplementary Information

Below is the link to the electronic supplementary material.

Supplementary Material 1 (DOCX 558 KB)

## Data Availability

No datasets were generated or analysed during the current study.

## Abbreviations

Abbreviation: Definition
Control: control cookies (no fat substitution)
T1: fat-reduced cookies formulated with native plantain flour
T2: fat-reduced cookies formulated with thermochemically modified plantain flour
TS: total starch
RDS: rapidly digestible starch i
SDS: slowly digestible starch
RS: resistant starch
C*: chromaticity
E-297: European food additive code (fumaric acid)
