# Supplementary material for: Partial Fat Substitution in Butter Cookies Using Thermochemically Modified Unripe Plantain (Musa paradisiaca AAB) Flour Treated with Fumaric Acid
Source: Plant Foods Hum Nutr. 2026 Aug 13;81(3):109. doi: 10.1007/s11130-026-01545-6 (PMC13473310; doi:10.1007/s11130-026-01545-6)
Supplement: Supplementary file 1 [file 11130_2026_1545_MOESM1_ESM.docx]

**Supplementary material section**

**Partial fat substitution in butter cookies using thermochemically modified unripe plantain (*Musa paradisiaca* AAB) flour treated with fumaric acid**

**Jhon Esteban Armenta-Roncancio^[1]^, Natalia José Muentes-Arismendy^[2]^, Eduardo Rodríguez-Sandoval^1^*, Jesús Humberto Gil-Gonzalez^1^, Leonardo Alexis Alonso-Gómez^[3]^**

**Keywords:** Low-fat foods, Food analysis, Bakery products, Food processing, Modified flour

**Graphical Abstract**

**Methodology**

**Materials**

Citrosan® (Tecnas, Colombia), fumaric acid (E-297; CAS No. 110-17-8; Addimentum S.A.S., Colombia), α-amylase (40 U/mL; Liquenzyme, Novozymes, Denmark), amyloglucosidase (50 U/mL; Optidex L-400, Danisco US Inc., Brazil), porcine pepsin (Merck, Germany), and D-glucose (Sigma-Aldrich, Germany) were used. All other reagents were of analytical grade.

**Unripe plantain flour preparation**

Unripe plantain fruits of the “Hartón” variety (*Musa paradisiaca* AAB) were used. The fruits were selected based on their physiological maturity (green, without signs of ripening) and purchased from a local market in Medellín, Colombia. They were sanitized by immersion in a Citrosan® solution (60 mg/L) for 15 min, peeled, and immersed in a citric acid solution (50 mg/L) for 10 min to minimize enzymatic oxidation [1]. Subsequently, the fruits were cut into 3-mm-thick slices and dehydrated in a forced-air oven (Memmert UF 450 Plus F, Germany) at 50°C for 6 h until a constant moisture content below 10% was reached. The dehydrated samples were milled in a hammer mill (IKA MF 10, Germany), sieved through a US No. 100 mesh (150 μm), and stored in double-sealed polypropylene airtight bags at −30°C in the dark until use.

**Modification by thermochemical treatment with fumaric acid**

A modified protocol described by Armenta et al. [2] was followed. Five hundred grams of unripe plantain flour were mixed with fumaric acid at 10% (w/w) relative to the flour. For every 100 g of unripe plantain flour, 200 mL of distilled water was added, and the resulting suspension was hydrated and stirred for 6 h at 25°C and 131 relative centrifugal force (RCF). Subsequently, thermochemical treatment was carried out in a forced-air oven at 140°C for 4 h following the methodology described by Alimi and Workneh [3]. The resulting mixture was washed with distilled water until pH 7 was reached. Finally, the samples were dehydrated at 50°C until the moisture content was below 10%, milled in a hammer mill, and sieved through a US No. 100 mesh.

**Preparation of control and low-fat cookies**

Control and low-fat cookies were prepared to evaluate the effect of partial fat substitution with unripe plantain flour. Three treatments were established:

- **Control:** Standard formulation using wheat flour and 100% of the original fat content, equivalent to 93 g of unsalted butter (7.69 kcal/g, 83% total fat) (Colanta, Medellín, Colombia), without plantain flour.
- **Treatment 1 (T1):** Formulation in which the butter content was reduced to 70 g (representing a 24.73% [w/w] fat reduction compared with the control) and replaced with an equivalent amount of native unripe plantain flour.
- **Treatment 2 (T2):** Formulation in which the butter content was reduced to 70 g (24.73% [w/w] fat reduction compared with the control) and replaced with an equivalent amount of thermochemically modified unripe plantain flour treated with fumaric acid.

The selected substitution level (70 g butter) was based on preliminary studies in which theoretical replacement levels of 5%, 15%, 25%, and 50% were evaluated. In those trials, substitution levels greater than the selected value adversely affected the textural and sensory properties of the cookies. Therefore, this formulation was selected as the optimal balance between fat reduction and sensory acceptability.

Cookie formulation and preparation were based on an adaptation of the method described by Cioccia [4]. Unsalted butter was mixed with powdered sugar for 2 min until a smooth and homogeneous cream was obtained. The creamed mixture was allowed to rest at 4°C for 15 min to stabilize. Subsequently, wheat flour, salt, powdered milk, plantain flour (native or modified), and egg were incorporated. The ingredients were mixed until a homogeneous dough was obtained without overkneading. The dough was rolled to a uniform thickness of 7.5 mm, cut into 50-mm-diameter circles, and baked in a convection oven (Bakerlux 6 Trays, Unox S.p.A., Italy) at 160°C. The cookies were baked for 10 min, after which the baking tray was rotated 180° and baking continued for an additional 2 min at the same temperature to ensure uniform baking. Finally, the cookies were cooled at 25°C for 15 min and, after reaching room temperature, were packaged in resealable polypropylene bags for storage.

**Physical properties of cookies**

The average diameter (Ø) was measured using a caliper (Non-Digital Calipers, 0.001 in/0.02 mm High Precision Durable Micrometer, China). Five consecutive cookies were aligned, and the average diameter was calculated using Equation (1) [4].

$$Ø =\frac{Length (5 cookies)}{5}$$

(1)

The average thickness was determined by measuring the height of five stacked cookies with the same caliper and calculating the average using Equation (2), according to Otero-Guzmán et al. [4].

$$Average height =\frac{Total height (5 cookies)}{5}$$

(2)

Weight loss during baking was calculated using Equation (3) as the percentage difference between the dough weight before and after baking.

$$Weight loss \left( \% \right)=\frac{before baking - after baking}{before baking}$$

(3)

The apparent volume of the cookies was estimated by assuming a cylindrical geometry using the measured diameter and thickness. The absolute volume was determined according to the method of Humeau Lab [5]. Briefly, cookies were crushed and transferred to a graduated cylinder. The sample was then subjected to 3,000 taps at a rate of 70 taps/min using a tap density tester (Professional Tap Tightness Tester, Dongguan Hongtuo Instrument Co., China). Porosity was calculated from the relationship between apparent and absolute volumes using Equation (4).

$$\mathrm{Porosity}\left( \% \right)= \frac{Apparent Volume - Absolute Volume}{Apparent Volume}$$

(4)

Moisture content was determined using a moisture analyzer (MB45, Ohaus, USA). One gram of sample was heated at 105°C until constant weight was achieved. All measurements were performed in triplicate. Water-holding capacity was determined according to the method of Paramasivam et al. [6] with minor modifications. One gram of crushed sample was vigorously mixed with 10 mL of distilled water and allowed to stand at room temperature for 24 h. The mixture was then centrifuged at 8,616 RCF for 15 min. The supernatant was decanted, and the wet residue was weighed. Measurements were performed in triplicate. Water activity ($a_{w}$) was measured at 25°C using a dew-point hygrometer (AquaLab 4TE Water Activity Meter; METER Group Inc., USA) with a sensitivity of 0.001 units. All measurements were performed in triplicate according to the method described by Otero-Guzmán et al. [4].

**Determination of proximate composition and pH**

Proximate composition was determined using standardized methods. Protein content was measured by the Kjeldahl method (AOAC 928.08, 1990). Fat content was determined using AOAC Method 945.38F. Ash content was determined according to ISO 2171:2023. Additionally, dough pH was measured using a pH meter (pH 211, HANNA Instruments, Woonsocket, RI, USA) according to AACC Method 02-52.01.

**Fracturability**

Fracturability, a key physical parameter for evaluating cookie quality, was determined using a TA-XT2i texture analyzer (Stable Micro Systems, Godalming, UK). The method described by Sánchez-Rivera et al. [7] was followed with minor modifications. The texture analyzer was equipped with a 50-kg load cell and a cylindrical P/2 probe (22 mm in diameter). The probe applied a single compression to the sample until 50% deformation was reached at a test speed of 1 mm/s. Ten measurements were performed, using a new cookie for each determination.

**Color**

Cookie color was evaluated by measuring the XYZ tristimulus values, which were subsequently converted to the CIE Lab* color space. This system uses three coordinates to describe color: L* (lightness), which represents brightness; a* (red-green axis), where positive values (+a*) indicate redness and negative values (−a*) indicate greenness; and b* (yellow-blue axis), where positive values (+b*) indicate yellowness and negative values (−b*) indicate blueness. Measurements were performed using a Minolta CR-400 colorimeter (Minolta Co., Ltd., Japan). From these coordinates, two additional indices were calculated: the whiteness index according to Equation (5), as proposed by Paramasivam et al. (2023), and chromaticity (C*) according to Equation (6), as proposed by Karaman et al. [8].

$$Whiteness index=100-\sqrt{\left( 100-L \right)+a^{2}+b^{2}}$$

(5)

$$C^{*}= \sqrt{a^{2}+b^{2}}$$

(6)

***In vitro* digestibility and susceptibility to enzymatic action of cookies**

Total starch content was quantified using the enzymatic method described by Ranathunga et al. [9] with minor modifications. Briefly, 200 mg of crushed cookie was mixed with 25 mL of sodium citrate-citric acid buffer (pH 5.2). The mixture was vigorously stirred for 30 s and heated in a water bath at 90°C. Subsequently, 100 μL of α-amylase (40 U/mL) was added, and hydrolysis was carried out at 90°C for 20 min with constant stirring at 70 rpm. The sample was then cooled to 60°C, 200 μL of amyloglucosidase solution (50 U/mL) was added, and enzymatic hydrolysis continued for 30 min at 60°C with constant stirring at 70 rpm. After hydrolysis, 2 mL of ethanol was added, and the sample was centrifuged at 7,000 RCF for 7 min. The supernatant was collected for reducing sugar determination.

Reducing sugars in the supernatant were quantified using the 3,5-dinitrosalicylic acid (DNS) method [10]. A 0.5-mL aliquot of the supernatant was diluted with distilled water at a 1:10 ratio. Subsequently, 1.5 mL of DNS solution was added, and the resulting mixture was heated in a water bath at 85°C for 10 min. After cooling, 5 mL of distilled water was added, and absorbance was measured at 540 nm using a spectrophotometer (Thermo Scientific Genesys 10 S UV-Vis, USA).

Rapidly digestible starch (RDS), slowly digestible starch (SDS), and resistant starch (RS) fractions were determined according to the method described by Wang et al. [11] with minor modifications. Briefly, 200 mg of cookie sample was weighed, and the lipid fraction was removed by hexane extraction for 4 h at 55°C with agitation (70 rpm). The protein fraction was hydrolyzed with 0.6 mL of pepsin solution (4,168 U/mL) at 37°C for 2 h with stirring (70 rpm) in a water bath. After pepsin hydrolysis, 15 mL of sodium citrate-citric acid buffer (pH 5.2) was added. The sample was incubated at 37°C, and 1 mL of enzyme solution (54 μL of α-amylase [350 U/g] and 6 μL of amyloglucosidase [140 U/g] diluted in pH 5.2 buffer) was added. To establish the digestion kinetics, enzymatic hydrolysis was carried out for 120 min. Every 20 min, an aliquot of the sample was collected and mixed with 2 mL of 70% ethanol, followed by centrifugation at 6,330 RCF for 7 min. The supernatant was diluted between 1:10 and 1:20 for reducing sugar analysis using the DNS method described above.

RDS and SDS were calculated using Equations (7) and (8), respectively. RS was calculated by difference according to Equation (9).

$$\mathrm{RDS}\left( \% \right)=\frac{\left( G20-G0 \right)\times0.9}{\mathrm{TS}} \times100$$

(7)

$$\mathrm{SDS}\left( \% \right)= \frac{\left( G120-GG20 \right)\times0.9}{\mathrm{TS}} \times100$$

(8)

$$\mathrm{RS}\left( \% \right)=100-RDS \left( \% \right)-SDS \left( \% \right)$$

(9)

where $G_{0}$is the amount of reducing sugars present in the cookies before enzymatic digestion (mg); $G_{20}$ and $G_{120}$ are the amounts of reducing sugars measured after 20 and 120 min of digestion, respectively, resulting from starch hydrolysis (mg); and TS is the total starch content (mg).

**Sensory acceptance**

A sensory acceptance test was conducted with 90 untrained consumers (18–50 years of age). The methodology followed the protocol described by Rakshit and Srivastav [13] with minor modifications. Participants were randomly selected to represent habitual cookie consumers. Using a randomized complete block design, each participant evaluated all three cookie formulations (Control, T1, and T2). Coded samples were presented simultaneously in randomized order to minimize bias, and water was provided between tastings as a palate cleanser. Consumers expressed their preferences using a three-point hedonic scale (“I like it,” “Indifferent,” or “I don't like it”). To determine whether the acceptance profile differed significantly among treatments, the data were analyzed by frequency analysis using Pearson’s chi-square ($x^{2}$) test for independence.

**Statistical analysis**

Statistical analyses were performed to compare the three cookie formulations. Physical properties were determined in quintuplicate (n = 5) and analyzed by analysis of variance. Significant differences among formulations were identified using Tukey’s honestly significant difference test at a significance level of *p* < 0.05. Sensory acceptance data were analyzed descriptively. All statistical analyses were performed using Statgraphics Centurion XVI (StatPoint Technologies, USA).

**Table and figures**

**Physicochemical properties of cookies**

Table 1 presents the physicochemical characteristics of baked products prepared with three treatments: Control (wheat flour with a conventional fat formulation), T1 (native plantain flour with fat reduction), and T2 (thermochemically modified unripe plantain flour treated with fumaric acid with fat reduction).

| Table 1. Physical, physicochemical, and proximate composition of reduced-fat butter cookies formulated with native and modified plantain flour as butter substitutes | | | |
| --- | --- | --- | --- |
| Property | Control | T1 | T2 |
| Diameter (cm) | 5.21 ± 0.19 ^a^ | 5.04 ± 0.12 ^a^ | 4.66 ± 0.09 ^b^ |
| Mass loss during baking (%) | 2.23 ± 0.06 ^a^ | 1.93 ± 0.12 ^b^ | 1.83 ± 0.08 ^b^ |
| Thickness (cm) | 1.22 ± 0.09 ^a^ | 1.22 ± 0.06 ^a^ | 0.9 ± 0.02 ^b^ |
| Expansion factor | 4.29 ± 0.43 ^b^ | 4.14 ± 0.27 ^b^ | 5.2 ± 0.15 ^a^ |
| Apparent volume (cm³) | 26.07 ± 2.02 ^a^ | 24.32 ± 0.95 ^a^ | 15.35 ± 0.67 ^b^ |
| Moisture content (g H₂O/100 g dry basis) | 3.93 ± 0.2 ^b^ | 5.14 ± 0.18 ^a^ | 3.88 ± 0.15 ^b^ |
| Water activity ($a_{w}$) | 0.43 ± 0.002 ^b^ | 0.52 ± 0.003 ^a^ | 0.39 ± 0.001 ^c^ |
| Water-holding capacity (g H_2_O/g dry basis) | 1.39 ± 0.21 ^a^ | 1.07 ± 0.22 ^a^ | 1.31 ± 0.02 ^a^ |
| Fat content (% w/w) | 30.26 ± 0.48 ^a^ | 23.34 ± 0.37 ^b^ | 23.44 ± 0.54 ^b^ |
| Dough pH | 7.12 ± 0.05 ^a^ | 7.08 ± 0.03 ^a^ | 6.85 ± 0.04 ^b^ |
| Protein (% w/w) | 6.19 ± 0.15 ^a^ | 5.22 ± 0.08 ^a^ | 6.35 ± 0.35 ^a^ |
| Ash (% w/w) | 0.37 ± 0.03 ^a^ | 0.41 ± 0.05 ^a^ | 0.44 ± 0.02 ^a^ |
| *Values with the same superscript letter within a row are not significantly different (Tukey’s HSD test, p < 0.05). Data are presented as mean ± SD (n = 5).* | | | |

**Color**

| Table 2. Fracturability and surface color parameters of baked products formulated with plantain flour | | | |
| --- | --- | --- | --- |
|  | Control | T1 | T2 |
| Fracturability (n) | 1.75 ± 0.05 ^c^ | 7.09 ± 0.72 ^a^ | 5.07 ± 0.73 ^b^ |
| L* | 74.49 ± 0.89 ^a^ | 64.62 ± 0.69 ^b^ | 61.93 ± 0.65 ^c^ |
| a* | 5.37 ± 0.26 ^b^ | 6.67 ± 0.46 ^a^ | 6.66 ± 0.31 ^a^ |
| b* | 23.30 ± 0.64 ^c^ | 30.77 ± 0.84 ^a^ | 27.19 ± 0.62 ^b^ |
| WI | 75.25 ± 0.48^a^ | 67.956 ± 0.91 ^c^ | 71.331 ± 0.66 ^b^ |
| C* | 23.73 ± 0.59^c^ | 31.48 ± 0.92 ^a^ | 27.99 ± 0.69 ^b^ |
| *Values with the same superscript letter within a row are not significantly different (Tukey’s HSD test, p < 0.05). Data are presented as mean ± SD (n = 5).* *L*, lightness; a*, red-green axis; b*, yellow-blue axis; WI, whiteness index; C*, chromaticity.* | | | |

Table 2 presents the color parameters and fracturability values of the control product and products formulated with plantain flour.

***In vitro* digestibility and starch digestion kinetics of cookies**

Table 3 presents the starch content and digestibility characteristics of low-fat cookies formulated with plantain flour.

| Table 3. Starch content and digestibility characteristics of low-fat plantain flour cookies | | | |
| --- | --- | --- | --- |
| Starch fraction (%) | Control | T1 | T2 |
| RDS | 45.44 ± 1.393 ^a^ | 42.69 ± 1.241 ^b^ | 44.26 ± 1.02 ^ab^ |
| SDS | 34.36 ± 3.016 ^a^ | 34.21 ± 5.718 ^a^ | 26.42 ± 2.696 ^b^ |
| RS | 20.19 ± 3.202 ^b^ | 23.1 ± 4.814 ^ab^ | 29.32 ± 3.661 ^a^ |
| *Values with the same superscript letter within a row are not significantly different (Tukey’s HSD test, p < 0.05). Data are presented as mean ± SD (n = 5).* | | | |

**Starch hydrolysis kinetics and modeling**

Figure 1. *Starch digestion kinetics of reduced-fat and control cookies as a function of time. The linear regression equation is expressed as y = ax + b, with the corresponding coefficient of determination (R²). Control (×, solid line): cookies prepared with wheat flour and a conventional fat formulation. T1 (▲, short dashed line): fat-reduced cookies formulated with native plantain flour. T2 (●, long dashed line): fat-reduced cookies formulated with thermochemically modified plantain flour. Data are presented as mean ± SD (n = 5).*

Figure 1 illustrates the progression of starch digestion by showing the variation in the amount of digested starch as a function of time.

Table 4 presents the kinetic model parameters for the control and reduced-fat products.

| Table 4. First-order kinetic model parameters for starch hydrolysis in bakery products. | | | |
| --- | --- | --- | --- |
| Parameter | Control | T1 | T2 |
| D(∞) | 65.07 | 60.19 | 44.86 |
| K (min⁻¹) | 0.475 | 0.468 | 0.334 |
| SUMSQ | 2525.85 | 2453.76 | 1243.98 |
| R^2^ | 0.8636 | 0.9298 | 0.8636 |
| Adjusted R² | 0.8364 | 0.9158 | 0.8364 |
| Durbin - Watson | 2.4765 | 2.8331 | 2.9355 |
| E | 8.9291 | 6.0857 | 6.2667 |
| RMSE | 19.966 | 13.608 | 14.013 |
| p-value | 0.00245 | 0.00045 | 0.00245 |
| F-value | 31.677 | 66.253 | 31.676 |
| *D(∞): glucose concentration at equilibrium (mg); K, first-order kinetic constant (min⁻¹); SUMSQ, sum of squared residuals; RMSE, root mean square error; R², coefficient of determination; E, standard error.* | | | |

**Sensory acceptance**

The frequency distribution analysis for sensory acceptance is presented in Figure 2.

*Figure 2. Sensory acceptance of reduced-fat bakery products formulated with plantain flour. Values represent the percentage of responses for each acceptance category. Sensory evaluation was conducted with 90 untrained panelists.*

*Chi-square statistic (*$x^{2}$*) = 126.67; degrees of freedom (df) = 4; p < 0.001; expected frequency (E) = 30.0; significance level (α) = 0.05*

**References**

[1]	Kaduji-Gadha R, Kaur R, Prasad K (2023) Effect of acid pretreatment on physicochemical, optical, functional, thermal, and morphological characteristics of unripe plantain and banana flour. J Food Chem Nanotechnol 9:147. [https://doi.org/10.17756/jfcn.2023-147](https://www.google.com/search?q=https://doi.org/10.17756/jfcn.2023-147)

[2]	Armenta-Roncancio JE, Rodriguez-Sandoval E, Gil-Gonzalez JH, Correa-Londoño GA (2026) Effect of heat treatment on the properties of plantain flour modified with fumaric acid. Starch Stärke 78:12. [https://doi.org/10.1002/star.70213](https://www.google.com/search?q=https://doi.org/10.1002/star.70213)

[3]	Alimi BA, Workneh TS (2018) Structural and physicochemical properties of heat moisture treated and citric acid modified acha and iburu starches. Food Hydrocoll 81:449–455. [https://doi.org/10.1016/j.foodhyd.2018.03.027](https://www.google.com/search?q=https://doi.org/10.1016/j.foodhyd.2018.03.027)

[4]	Otero-Guzmán N, Cadena-Chamorro E, Ciro-Velásquez HJ, Mejía-Villota A, Rodriguez-Sandoval E (2023) Application of modified cassava starch as a fat substitute in cracker production. J Food Process Preserv 2023:6888180. [https://doi.org/10.1155/2023/6888180](https://www.google.com/search?q=https://doi.org/10.1155/2023/6888180)

[5]	Humeau Lab (2025) Tap Density Tester User Manual. <https://www.humeau.com/media/blfa_files/__7700_Gebrauchsanleitung_ENG__206371.pdf>

[6]	Paramasivam SK, Subramaniyan P, Thayumanavan S, Shiva KN, Narayanan S, Raman P, Subbaraya U (2023) Influence of chemical modifications on dynamic rheological behaviour, thermal techno-functionalities, morpho-structural characteristics and prebiotic activity of banana starches. Int J Biol Macromol 249:126125. [https://doi.org/10.1016/j.ijbiomac.2023.126125](https://www.google.com/search?q=https://doi.org/10.1016/j.ijbiomac.2023.126125)

[7]	Sánchez-Rivera MM, Bello-Pérez LA, Tovar J, Martinez MM, Agama-Acevedo E (2019) Esterified plantain flour for the production of cookies rich in indigestible carbohydrates. Food Chem 292:1–5. [https://doi.org/10.1016/j.foodchem.2019.04.007](https://www.google.com/search?q=https://doi.org/10.1016/j.foodchem.2019.04.007)

[8]	Karaman K, Pinar H, Ciftci B, Kaplan M (2025) Effects of banana flour on some physicochemical, textural, bioactive, and sensory properties of gluten-free cookie. Food Sci Nutr 13:e4756. [https://doi.org/10.1002/fsn3.4756](https://www.google.com/search?q=https://doi.org/10.1002/fsn3.4756)

[9]	Ranathunga SD, Kalscheur KF, Anderson JL, Herrick KJ (2017) Evaluation of a modified method to measure total starch in animal feeds. Anim Feed Sci Technol 226:124–132. [https://doi.org/10.1016/j.anifeedsci.2017.01.020](https://www.google.com/search?q=https://doi.org/10.1016/j.anifeedsci.2017.01.020)

[10]	Deshavath NN, Mukherjee G, Goud VV, Veeranki VD, Sastri CV (2020) Pitfalls in the 3, 5-dinitrosalicylic acid (DNS) assay for the reducing sugars: Interference of furfural and 5-hydroxymethylfurfural. Int J Biol Macromol 156:180–185. <https://doi.org/10.1016/j.ijbiomac.2020.04.045>

[11]	Wang W, Liu T, Zhi W, Zhou Y, Hu A, Zheng J (2022) Study on the preparation and digestibility of malic acid sweet potato starch ester under microwave assistance. Starch Stärke 74:2200011. [https://doi.org/10.1002/star.202200011](https://www.google.com/search?q=https://doi.org/10.1002/star.202200011)

1. Department of Agricultural and Food Engineering, Faculty of Agricultural Sciences, Universidad Nacional de Colombia - Medellín Campus, Colombia
2. Universidad Nacional de Colombia – Medellín Campus – Faculty of Mines – Department of Processes and Energy – Av. 80 #65 - 223, Villa Flora, Medellín, Colombia
3. Universidad de los Llanos, Grupo de Investigación “Ciencia, Tecnología e Innovación Agroindustrial (CITIA)”, Km. 12 Vía Puerto López. Villavicencio, Colombia.

   *** Corresponding author: +57 6044309154. edrodriguezs@unal.edu.co**
